# BL09XU: an advanced hard X-ray photoelectron spectroscopy beamline of SPring-8

**DOI:** 10.1107/S160057752300629X

**Published:** 2023-08-23

**Authors:** Akira Yasui, Yasumasa Takagi, Taito Osaka, Yasunori Senba, Hiroshi Yamazaki, Takahisa Koyama, Hirokatsu Yumoto, Haruhiko Ohashi, Koji Motomura, Kyo Nakajima, Michihiro Sugahara, Naomi Kawamura, Kenji Tamasaku, Yusuke Tamenori, Makina Yabashi

**Affiliations:** a Japan Synchrotron Radiation Research Institute, 1-1-1 Kouto, Sayo, Hyogo 679-5198, Japan; b RIKEN SPring-8 Center, 1-1-1 Kouto, Sayo, Hyogo 679-5148, Japan; ESRF – The European Synchrotron, France

**Keywords:** hard X-ray photoelectron spectroscopy, HAXPES, beamlines, X-ray optics, high-flux microbeams, double-crystal X-ray phase retarders

## Abstract

The BL09XU beamline of SPring-8 has been reorganized into a beamline dedicated for hard X-ray photoelectron spectroscopy (HAXPES) to provide advanced capabilities with upgraded optical instruments. The beamline has two HAXPES analyzers to cover a wide range of applications. Two sets of double channel-cut crystal monochromators with the Si(220) and (311) reflections were installed to perform resonant HAXPES analyses with a total energy resolution of less than 300 meV over a wide energy range (4.9–12 keV) while achieving a fixed-exit condition. A double-crystal X-ray phase retarder using diamond crystals controls the polarization state with a high degree of polarization over 0.9 in the wide energy range 5.9–9.5 keV. Each HAXPES analyzer is equipped with a focusing mirror to provide a high-flux microbeam. The design and performance of the upgraded instruments are presented.

## Abstract 2

The BL09XU beamline of SPring-8 has been upgraded to a beamline dedicated for hard X-ray photoelectron spectroscopy to provide advanced capabilities with improved optical instruments.

## 1. Introduction

Photoelectron spectroscopy is a powerful method for investigating the electronic states of materials, and has been recognized as an indispensable tool for a broad range of research fields, from basic science to industrial applications. In particular, hard X-ray photoelectron spectroscopy (HAXPES), in which a hard X-ray beam is used to generate photoelectrons, has a unique capability to investigate the bulk electronic state distribution (Lindau *et al.*, 1974 ▸; Fadley, 2010 ▸; Woicik, 2016 ▸). The depth range that can be measured in HAXPES is several tens of nanometres from the surface (about 20 nm at 8 keV), which is inaccessible in conventional photoelectron spectroscopy with vacuum ultraviolet or soft X-ray beams (Tanuma *et al.*, 2003 ▸; Sacchi *et al.*, 2005 ▸). HAXPES analyses, however, have been impractical because of the small photoionization cross-section in the hard X-ray region, which is two orders of magnitude lower than that for the Al *K*α X-ray source (1.5 keV) (Yeh & Lindau, 1985 ▸). The advent of brilliant synchrotron radiation sources triggered a paradigm shift for HAXPES, as shown in the rapid growth in the early 2000s (Takata *et al.*, 2005 ▸; Kobayashi *et al.*, 2003 ▸). The advances in HAXPES techniques combined with advanced X-ray optics, such as high-resolution monochromators (HRMs), X-ray phase retarders (XPRs) and X-ray focusing optics, have enabled detailed studies of chemical bonding and microanalysis of electronic states (Kalha *et al.*, 2021 ▸).

Nowadays, HAXPES is one of the most popular methods in synchrotron light sources. The specifications for HAXPES beamlines worldwide in 2020 are summarized by Kalha *et al.* (2021 ▸). At SPring-8 in Japan, twelve HAXPES analyzers have been installed in various beamlines including public beamlines BL09XU, BL47XU (Ikenaga *et al.*, 2013 ▸, 2018 ▸) and BL46XU (Yasuno *et al.*, 2016 ▸). However, they were not fully dedicated to HAXPES, and frequent switching of beamline instruments was inevitable, causing a considerable loss of efficiency.

To overcome these problems, we decided to refurbish BL09XU and BL46XU into beamlines dedicated for HAXPES. As a first step, we reorganized BL09XU in 2021 with integrating HAXPES activities in BL09XU and BL47XU to perform advanced HAXPES measurements, such as resonant analysis and three-dimensional microanalysis. In particular, resonant HAXPES reveals electronic structures with enhanced element- and valence-selectivity by scanning the incident X-ray energy near the X-ray absorption edges (Ikenaga *et al.*, 2018 ▸). A typical target is the quantum critical phenomena of valence fluctuation through the 2*p*–5*d* resonant HAXPES of rare-earth compounds (Maeda *et al.*, 2020 ▸). Furthermore, combining the polarization control of incident X-rays with the energy-scanning capability, one can also extract electron orbital symmetry (Fujiwara *et al.*, 2016 ▸; Mori *et al.*, 2014 ▸) and magnetic information (Ouardi *et al.*, 2011 ▸; Kozina *et al.*, 2011 ▸; Ueda *et al.*, 2019 ▸).

As key optical devices for the advanced HAXPES analyses, we installed double channel-cut crystal monochromators (DCCMs) and a double-crystal X-ray phase retarder (DXPR) in the optics hutch (OH). So far, single channel-cut crystal monochromators (CCMs) were utilized at both BL09XU and BL47XU. The range of energy scans for resonant HAXPES was, however, limited practically within ∼100 eV because of the lateral shift of the beam height during the scan. On the other hand, a fixed-exit condition can be fulfilled for a DCCM arranged in the (+, −, −, +) geometry, as the beam axis lifted by the first (+, −) CCM is compensated by the second (−, +) CCM. The second key device, DXPR, converts the horizontally linear polarized incident beam into a circular or vertically linear polarized one. With a conventional single-crystal XPR, the degree of polarization state decreases with increased photon energies, due to the phase-shift inhomogeneity caused by the finite angular divergence and bandwidth of the incident X-rays (Okitsu *et al.*, 2001 ▸). In the case of the DXPR, the phase-shift inhomogeneity in the first XPR crystal can be cancelled out by the second crystal (Scagnoli *et al.*, 2009 ▸) and, consequently, a high degree of polarization state can be obtained over a wide energy range.

In the experimental hutches (EHs), we installed two HAXPES analyzers – a high-resolution type and a wide-acceptance-angle type – and focusing mirrors that match the performance of the analyzers. A new instrument control platform was installed for integrated management of HAXPES analyzers and beamline optical components. In this paper, we introduce the design and performance of BL09XU, and provide two example studies: a resonant HAXPES measurement and a three-dimensional spatially resolved electronic structure analysis.

## 2. Design

**Figure 1:**
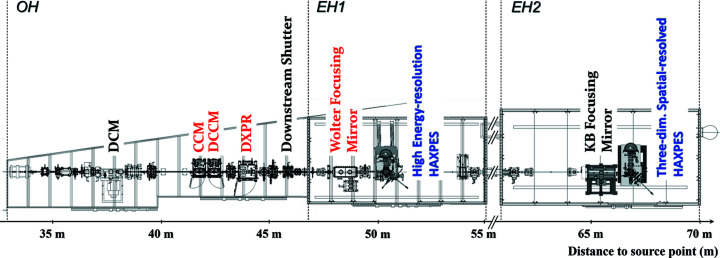
Beamline layout of BL09XU. All optical components except for the focusing system are located in the OH. Two sets of photoelectron analyzers and focusing mirrors with different specifications are installed in EH1 and EH2, respectively.

BL09XU is a standard hard X-ray beamline with a 4.5 m in-vaccum linear undulator with a period length of 32 mm (Hara *et al.*, 1998 ▸; Kitamura, 1998 ▸). The beamline layout is shown in Fig. 1 ▸. In the OH, a liquid-nitro­gen-cooled double-crystal monochromator (DCM) with the Si(111) reflection is employed as a high-heat-load monochromator. For HRMs, two DCCMs and a CCM are installed. The DXPR system is located downstream of the HRMs. Since a downstream shutter (DSS) is located at the end of the OH, the heat loads to the HRMs and the DXPR are kept constant, even when entering the EH to, for example, exchange samples.

Two photoelectron analyzers with different specifications are installed in each of the two EHs arranged in tandem. The HAXPES system and focusing mirror of EH1 can be easily retracted from the optical axis for transporting the beam to EH2. In addition, we are now able to access the EH1 instrument for experimental preparation and maintenance while another experiment is performed in downstream EH2. This operation mode is called ‘access mode’. This allows, for example, time-consuming sample processing to be performed and sample transfer to the measurement chamber in EH1 in advance. Users can start measurements as soon as X-rays are introduced into the EH1 instrument. For focusing mirrors, a Wolter mirror and a Kirkpatrick–Baez (KB) mirror are installed in EH1 and EH2, respectively. Their detailed designs are introduced below.

### 2.1. HRMs

**Figure 2:**
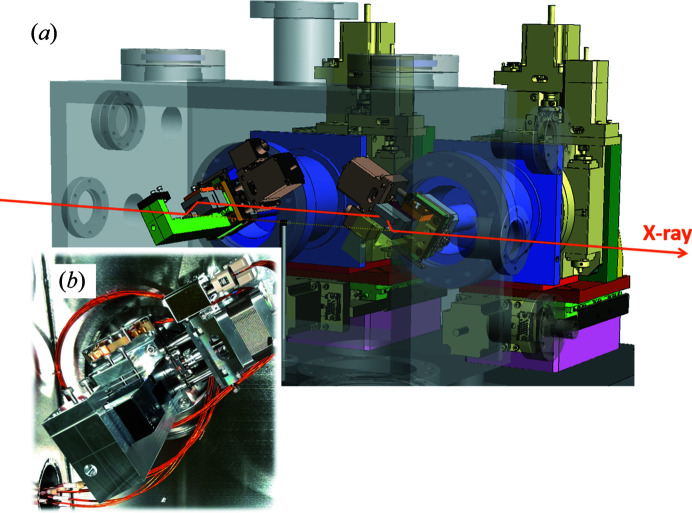
(*a*) Schematic and (*b*) photograph of the DCCM system. In (*a*), the vacuum chamber is displayed translucently without showing one of two crystals to improve the viewability of the control mechanics. The switching of the two crystals is performed by horizontally translating the stages loaded with upper and lower bounce crystals. In-plane rotation mechanisms of crystals are equipped to avoid simultaneous reflections on different crystal planes. A beam stopper is installed between the two crystals to cut off X-rays with higher energies.

For HRMs, two DCCMs and a CCM are installed. They are arranged in tandem along the optical axis and can be easily switched by horizontal translations. Fig. 2 ▸ shows the DCCM system to be installed. The DCCMs with low-order reflections of Si(220) or (311) provide a high-intensity beam with a moderate resolution over the wide energy range 4.9–12 keV, and are used for a wide range of applications including resonant HAXPES. The Bragg angle of the crystals can be set from 18° to 45°. The Bragg angles, photon energies (*h*ν) and energy resolutions (Δ*E*_M_/*h*ν; where Δ*E*_M_ is the energy bandwidth) for Si(220) and (311) reflections are summarized in Table 1 ▸. Δ*E*_M_/*h*ν is ∼3.8 × 10^−5^ and ∼1.6 × 10^−5^ for Si(220) and (311), respectively.

On the contrary, CCM is used for high-resolution analysis with a fixed Bragg angle of 85° and discrete photon energies. Δ*E*_M_/*h*ν is 7.5 × 10^−6^ at 5.95 keV with Si(333), 4.5 × 10^−6^ at 7.94 keV with Si(444) and 1.6 × 10^−6^ at 9.92 keV with Si(555).

### 2.2. DXPR

**Figure 3:**
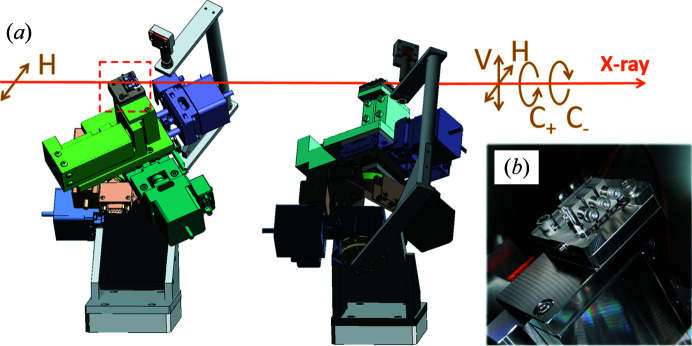
(*a*) Schematic of DXPR mechanics. In-plane rotation mechanisms of crystals are equipped to avoid simultaneous reflections on different crystal planes. (*b*) Photograph of a holder for mounting diamond crystals. The switching of crystals is performed by translating them in the orthogonal direction to the beam.

Fig. 3 ▸(*a*) shows the DXPR system in a vacuum chamber, which is similar to the system installed at BL19LXU of SPring-8 (Fujiwara *et al.*, 2016 ▸). Three pairs of diamond (001) crystals with thicknesses of 0.1, 0.2 and 0.4 mm operating in the 220 Laue geometry have been installed. All the diamond crystals have a strain-relief structure and are mechanically mounted on the dedicated holders for better stability and good compatibility with ultra-high-vacuum environments [Fig. 3 ▸(*b*)]. These three pairs cover a wide energy range (4.9–12 keV) while keeping a high degree of polarization >0.9 and a transmission >0.2, as shown in Table 2 ▸.

### 2.3. Focusing mirrors

**Figure 4:**
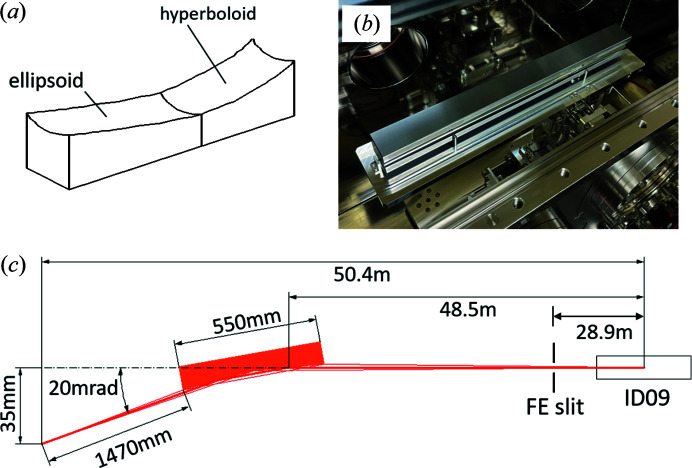
(*a*) Conceptual figure and (*b*) photograph of the Wolter focusing mirror. This mirror has an elliptical and a hyperbolic part on a single substrate, and is installed in a vacuum chamber. (*c*) Top-view optical layout of the Wolter mirror system. The long working distance of 1470 mm allows a large degree of freedom in the layout of components such as X-ray detectors and cameras for sample observation in the HAXPES instrument.

A monolithic Wolter mirror, which has an elliptical and a hyperbolic surface shape on a single substrate, was adopted for the focusing system of EH1 (Senba *et al.*, 2020 ▸, 2023 ▸). A schematic of the mirror is shown in Fig. 4 ▸(*a*). In Fig. 4 ▸(*b*), the mirror is installed in an ultra-high-vacuum chamber to minimize contamination on its surface. A typical focused beam size is designed to 0.5 µm (V) × 26 µm (H) when fully open with a primary slit in the front-end section (FE slit) located 28.9 m from the undulator source [Fig. 4 ▸(*c*)]. As an important characteristic, this mirror has a high angular tolerance of about ±400 µrad to the pitching error, which is much larger than that of KB-type mirrors (a few microradians), facilitating realignment procedures after switching instruments with enhanced stability. This mirror can be moved 300 mm in the upstream direction of the optical axis so as to enlarge the beam size at the sample position. This is effective in suppressing charge-up when measuring photoelectrons on low-conductivity samples.

At EH2, a KB focusing mirror system is installed (Ikenaga *et al.*, 2018 ▸). A focused beam size of 1 µm × 1 µm is achievable by limiting the horizontal aperture width of the FE slit. Both mirror systems have a large focal length, which allows large flexibility for the use of the HAXPES systems. Key design parameters are summarized in Table 3 ▸.

These focusing mirrors as well as the monochromators are useful to reduce the fraction of the harmonic radiation from the undulator source, which is negligible in most cases but sometimes generates unwanted signals in HAXPES measurements. For example, the fraction of the third harmonic should be less than 0.1% at a fundamental of 4.9 keV with the Si(220) DCCM and the Wolter mirror. This fraction decreases when we use another HRM and/or higher fundamental energies. Note that the second harmonic is well excluded at the Si(111) DCM because the Si(222) reflection is forbidden.

### 2.4. HAXPES instruments

**Figure 5:**
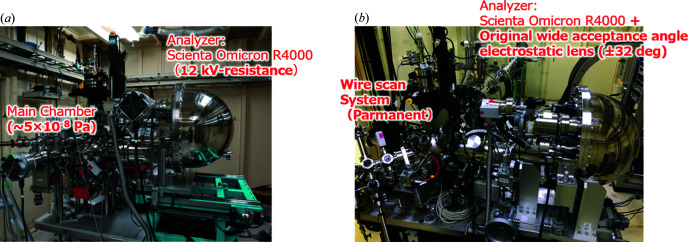
HAXPES instruments installed in (*a*) EH1 and (*b*) EH2. (*a*) The EH1 analyzer detects photoelectrons with kinetic energies up to 12 keV. (*b*) A wide-acceptance-angle objective lens is equipped in front of the analyzer in EH2, enabling a photoelectron detection angle of ±32°.

Two SCIENTA OMICRON R4000 HAXPES analyzers are installed in EH1 and EH2, as shown in Fig. 5 ▸. In EH1, an analyzer to detect high-kinetic-energy photoelectrons up to 12 keV allows us to analyze electronic states in deeper buried interfaces, as well as to perform resonant HAXPES of 5*d* transition elements with *L*-absorption edges in *h*ν = 10–12 keV. Therefore, resonant HAXPES has the potential to selectively extract the contribution of 5*d* electrons in the valence band, which directly contribute to the physical properties. On the other hand, a specifically designed wide-acceptance-angle objective lens is mounted in front of another analyzer in EH2, enabling a photoelectron detection angle of ±32° (Ikenaga *et al.*, 2013 ▸, 2018 ▸). The feature is suitable for simultaneous acquisition of depth information of chemical bonding states from the sample surface to the bulk, which is important especially for local analysis using the 1 µm focused beam. This analyzer was relocated from BL47XU.

The EH1 instrument is mainly used for fundamental physics research related to strongly correlated electron systems and other materials. The system is equipped with a permanently installed silicon drift detector (SDD). In resonant HAXPES measurements, the SDD can be used not only to determine the absorption edge energy, but also to simultaneously acquire electronic states at different detection depths by simultaneous measurement of HAXPES and fluorescence. On the other hand, EH2 is mainly used for applied materials research, taking advantage of its analyzer and mirror features. In particular, *in situ* voltage-applied measurements are possible using a sample manipulator with four-terminal electrodes. Direct observation of band-bending in semiconductor and dielectric materials has been performed using this manipulator and the wide acceptance angle analyzer. Both instruments are capable of surface treatment of samples in the preparation chamber using a fracture tool and a file, and the sample temperature can be set from 20 to 400 K by liquid helium flow. In addition, a portable sample-preparation chamber with an Ar or a radio frequency sputtering and sample heating to about 800 K is available. It can be connected to either HAXPES instrument, and the treated sample can be transferred to the measurement chamber without exposure to the atmosphere.

### 2.5. Instrument control method

**Figure 6:**
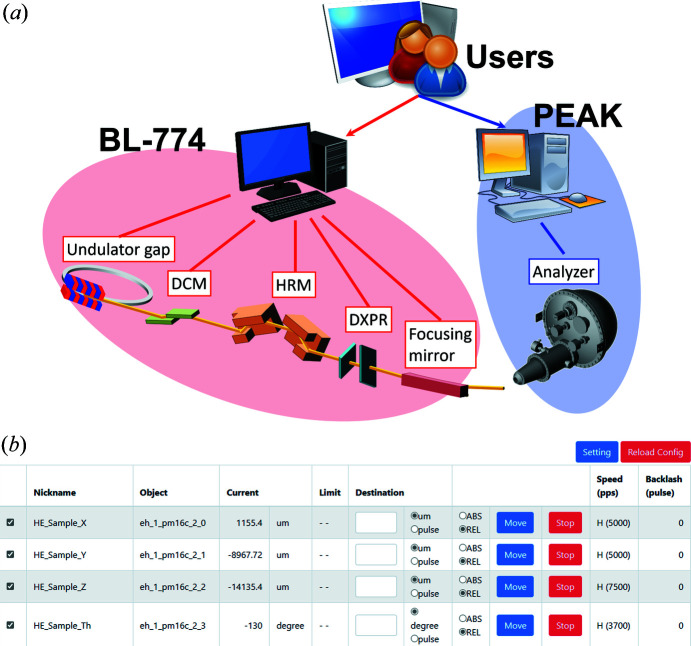
(*a*) Conceptual diagram of the instrument control using the BL-774 and PEAK systems. All devices in BL-774 are controlled via TCP/IP. (*b*) Screenshot of a web-based BL-774 software, performing device control and communication management.

For conducting experiments efficiently, the instrument control system has been updated. All the optical instruments and stages of the HAXPES instruments are controlled by the instrument control platform ‘BL-774’ (Nakajima *et al.*, 2022 ▸). This is a Python-based system that allows for the control of different types of instruments, such as motor-driven devices and piezo stages, in a unified manner. Fig. 6 ▸(*a*) shows a conceptual diagram of the instrument control using the BL-774 system. Device control and communication management are carried out by web-based software as shown in Fig. 6 ▸(*b*). On the other hand, a SCIENTA OMICRON ‘*PEAK*’ software is introduced for control of the analyzer and HAXPES measurements. This software, built in Python, allows integration with the BL-774 system. Note that all the instruments in the beamline can be basically controlled from outside of the SPring-8 site to conduct remote experiments.

## 3. Performance

The typical performance of the systems is summarized in this section.

### 3.1. Energy resolution

**Figure 7:**
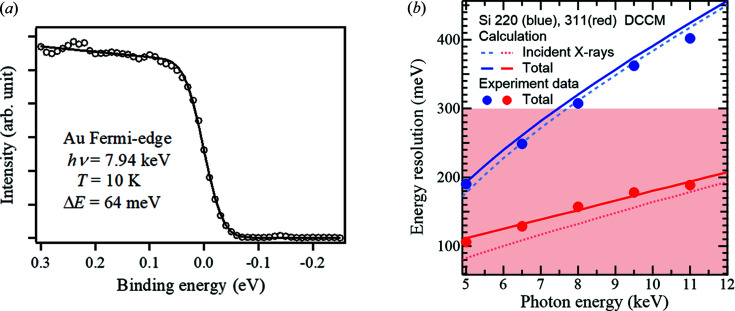
(*a*) Fermi-edge profile of a gold sample obtained using Si(444) CCM at the photon energy 7.94 keV. Open circles indicate the experimental data and the solid line represents the fitting result of them. (*b*) Total energy bandwidths as a function of the photon energy and the index of the DCCM crystal. The solid circles indicate those obtained by Fermi-edge measurements of a gold sample, and the solid lines show the calculated values. The dotted lines are the theoretical energy bandwidths of the incident X-rays after DCCMs.

The total energy bandwidth, Δ*E*_T_, was evaluated using the upgraded optical instruments and the photoelectron analyzer in EH1. Δ*E*_T_ is obtained by the convolution of Δ*E*_M_ and the energy bandwidths of the photoelectron analyzer, Δ*E*_A_, *i.e.* Δ*E*_T_ = (Δ*E*_M_^2^ + Δ*E*_A_^2^)^1/2^. First, we evaluated the typical energy resolution at *h*ν = 7.94 keV using the Si(444) CCM and an analyzer pass-energy, *E*_P_, of 50 eV. Fig. 7 ▸(*a*) shows a Fermi-edge profile of a gold sample at 10 K. Δ*E*_T_ was evaluated to be 64 meV full width at half-maximum from the fittings of the profile. This energy resolution is reasonable compared with the values previously reported (Takata *et al.*, 2005 ▸; Ueda, 2013 ▸).

Next, we evaluated the energy resolution at *E*_P_ = 100 eV over the wide photon energy range. Under this analyzer condition, Δ*E*_T_ was obtained from Fermi-edge measurement of the gold sample at 20 K for both Si(220) and Si(311) DCCMs in the energy range 5.0–11 keV. Fig. 7 ▸(*b*) shows Δ*E*_T_ as a function of the incident X-ray energy for the Si(220)/(311) DCCMs. The solid lines show Δ*E*_T_ calculated using Δ*E*_M_ from analyses with the DuMond diagrams considering the angular divergence of the incident beams (dashed lines) and a theoretical analyzer resolution, Δ*E*_A_ = *wE*_P_/2*R* = 75 meV, where *w* = 0.3 mm and *R* = 200 mm are the slit width and the radius of the analyzer, respectively. For both Si(220) and Si(311) DCCMs, the experimental Δ*E*_T_ were in good agreement with the calculated values. With the Si(311) DCCM, photoelectron measurements with a high energy resolution of less than 200 meV are achievable over the entire region. On the other hand, the Si(220) DCCM has a relatively modest resolution whereas the X-ray flux is approximately four times higher than that with the Si(311) DCCM. Therefore, efficient measurements are possible by selecting an adequate DCCM for individual applications.

### 3.2. Degree of polarization

The polarization degree with the XPRs with a thickness of 0.2 mm was measured in the energy range from 5.9 keV to 9.5 keV in linear and circular polarization. The degree of linear polarization, *P*_L_, was evaluated from the X-ray intensity scattered vertically and horizontally with a thin film of glassy carbon placed downstream of the XPRs. The degree of circular polarization, *P*_C_, was estimated by combining the experimental values of *P*_L_ with the theoretical calculations.

**Figure 8:**
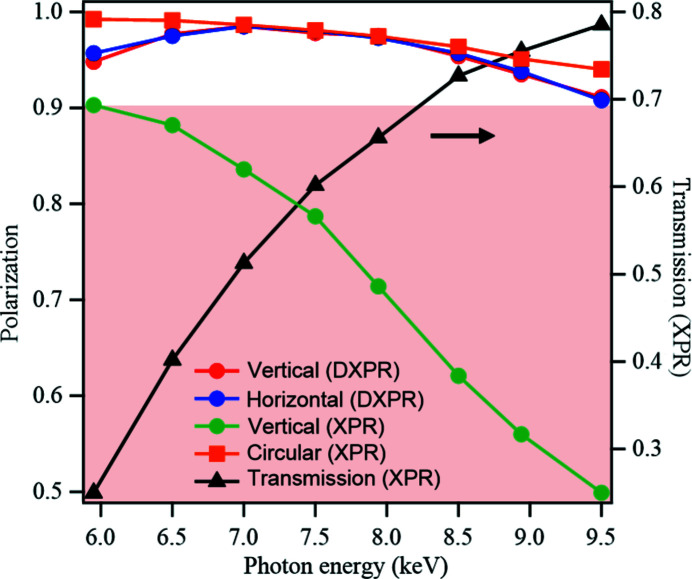
Absolute values of the degree of linear and circular polarizations as a function of the photon energy for XPRs of 0.2 mm thickness in *h*ν = 5.9–9.5 keV. The right-hand axis indicates the transmission intensity of X-rays through a single XPR.

Fig. 8 ▸ shows energy dependences of *P*_C_ and *P*_L_. For the circular polarization, a single XPR was used, and a sufficiently high *P*_C_ above 0.9 was obtained over the entire photon energy range. For the linear polarization, we measured *P*_L_ with single and double XPRs. For the vertical polarization with single XPR, a high *P*_L_ of about 0.9 was obtained at 5.9 keV, but a significant decrease in *P*_L_ was observed for the higher photon energy range. On the other hand, high *P*_L_ values above 0.9 were obtained for the DXPR over the entire region. A similar result was obtained for the horizontally polarized condition. We confirmed that good performance was achieved as designed. The advantage of using double XPRs to obtain a horizontally polarized beam is that the transmittance of the beam is almost the same as that of the vertically polarized beam in linear dichroism measurements in which the vertical and horizontal polarization is switched, and the polarization direction can be switched quickly by changing the angle of the second XPR.

The X-ray transmissions with the single XPR are shown on the right-hand axis. Those of double XPRs are derived by squaring these values. The transmission is about 0.8 at 9.5 keV, but decreases significantly as the energy decreases. At 5.9 keV, the transmission for the single XPR is ∼0.25, and thus it is ∼0.063 for the DXPR. Therefore, the number of XPRs should be selected based on the degree of polarization and the transmission required for the measurement. To maintain the high degree of polarization in the wider energy range (*h*ν = 4.9–12 keV) and simultaneously use a beam with increased transmittance, we introduced double XPRs with thicknesses of 0.1 mm and 0.4 mm.

### 3.3. Focused beam size and flux at sample

We evaluated the focused beam size of the Wolter mirror in EH1 using a knife-edge scan with a 200 µm-diameter gold wire. The size of the focused beam at the sample position is measured to be 1.3 µm (V) and 21 µm (H). The high beam flux of 5.6 × 10^12^ photon s^−1^ is obtained using the Si(311) DCCM at *h*ν = 7.94 keV. Furthermore, we confirmed that this focusing performance is reproduced once the mirror is retracted and re-installed. By moving the mirror 200 mm in the upstream direction, the beam size can be enlarged to about 70 µm both vertically and horizontally. The beam density decreases by a factor of about 150 while maintaining the beam intensity, which is very effective in suppressing charge-up in samples for photoelectron measurements.

**Figure 9:**
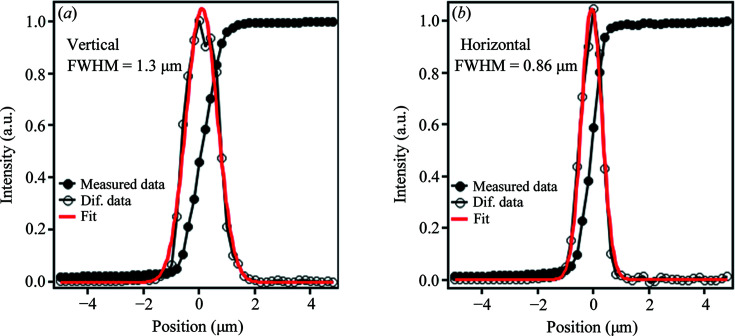
Beam intensity profiles focused using the KB mirror and their differential intensity profiles in the (*a*) horizontal and (*b*) vertical directions.

We also evaluated the focused beam size for the KB mirror in EH2 using the same method as for the Wolter mirror. The beam size at the sample position is determined to be 1.5 µm (V) × 11 µm (H) with a beam flux of 6.3 × 10^12^ photon s^−1^ using an Si(311) DCCM at *h*ν = 7.94 keV. This high-brilliance microbeam enables efficient photoelectron collection, and the photoelectron intensity is about 200 times higher than that at BL47XU. By narrowing the horizontal width of the FE slit to 0.03 mm, a micro-focused beam of size 1.3 µm (V) × 0.86 µm (H) is obtained on the sample, as shown in Fig. 9 ▸, with a beam flux of 1.3 × 10^11^ photons s^−1^. Note that the beam intensity is about 15 times higher than that of the previous beam available at BL47XU (Ikenaga *et al.*, 2013 ▸).

The parameters obtained are summarized in Table 4 ▸. Compared with Table 3 ▸, the horizontal focused beam sizes for both the Wolter and KB mirrors are slightly smaller except for that in the condition of FE width = 33 µm in EH2. We consider that the horizontal beam size at the emission point is about 80% smaller than expected. On the other hand, the vertical ones are larger than the designed values. This is considered to be due to fluctuations of the incident beam, vibrations of the mirrors *etc*.

### 3.4. HAXPES analyzer

**Figure 10:**
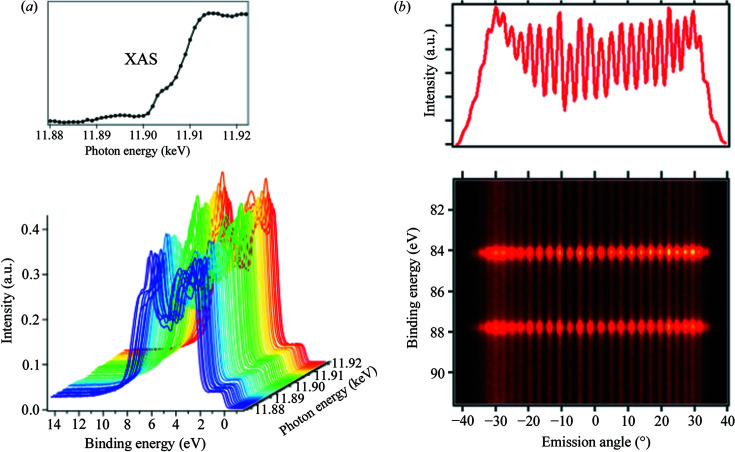
(*a*) Resonant HAXPES spectra of the valence band at the Au *L*_3_ edge (*h*ν ≃11.9 keV) for a gold sample obtained using the EH1 analyzer at *E*_P_ = 100 eV. The upper graph is an X-ray absorption spectrum of this sample. (*b*) Angular profile of an Au 4*f* AR-HAXPES spectrum for the angular test device. The number of peaks indicate that the EH2 analyzer has photoelectron acceptance angles of larger than ±32° because the period of the slits is 2.8°.

The EH1 analyzer is capable of measuring photoelectrons up to 12 keV, and thus enables the user to perform resonant HAXPES measurements of 5*d* electron-based materials. Fig. 10 ▸(*a*) shows resonant HAXPES spectra of the valence band in the Au *L*_3_-edge at *h*ν ≃ 11.9 keV for a gold sample. The X-ray absorption spectrum of the same sample at the Au *L*_3_-edge obtained using a SDD is displayed in the upper panel. We confirmed that resonant measurements in the valence band, predominantly the Au 5*d* band, are feasible.

The analyzer at EH2 is characterized by acquiring photoelectrons with a wide emission angle of ±32°. The angular dependence of the photoelectron intensity in the angle-resolved (AR) mode was evaluated using an angular test device (Ikenaga *et al.*, 2013 ▸). Fig. 10 ▸(*b*) shows an angular profile of an AR-HAXPES spectrum at the Au 4*f* core level at *E*_P_ = 200 eV. The number of peaks indicates that the analyzer has photoelectron acceptance angles larger than ±32°.

## 4. Applications

### 4.1. Resonant HAXPES (EH1)

**Figure 11:**
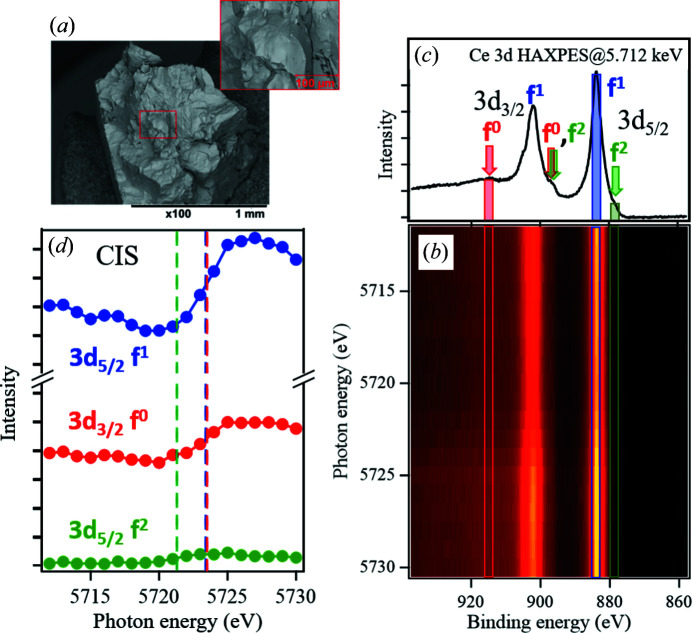
(*a*) Scanning electron microscopy (SEM) images of a fractured CePd_2_Si_2_ polycrystalline sample. (*b*) Intensity plot of Ce 3*d* resonant HAXPES spectra at the Ce *L*_3_ edge. (*c*) Ce 3*d* HAXPES spectrum at a non-resonant condition (*h*ν = 5.712 keV). (*d*) Constant initial state spectra for 3*d*_3/2_ *f* ^0^ (binding energy, *E*_B_ = 913.8 eV), 3*d*_5/2_ *f* ^1^ (*E*_B_ = 882.7 eV) and 3*d*_5/2_ *f* ^2^ peaks (*E*_B_ = 877.3 eV).

As an example for resonant HAXPES analyses, we performed measurements on a polycrystalline CePd_2_Si_2_ sample at 20 K using beams with the focused size 1.3 µm × 21 µm at the sample position. The fractured surface of this sample is shown in Fig. 11 ▸(*a*). Due to the roughness of the surface, the photoelectron intensity could drastically fluctuate if the beam irradiation position were varied with energy sweeping. Ce 3*d* resonant HAXPES spectra of this sample at the Ce *L*_3_-absorption edge are shown in Fig. 11 ▸(*b*). Fig. 11 ▸(*c*) shows the Ce 3*d* spectrum under a non-resonant condition, and the constant-initial-state (CIS) spectra at the binding energies indicated by the arrows are shown in Fig. 11 ▸(*d*). In the CIS spectra, intensity evolution associated with Ce 2*p*–5*d* resonant excitation is clearly observed. This spectral shape is explained by Fano (1961 ▸), which is the result of quantum mechanical interference between different transition processes having the same final state in the resonance process. Because of the fixed-exit condition and the stability on intensity of the incident beam during the energy sweeping, CIS spectra are now acquired with a very high signal-to-noise ratio, allowing the resonant energies, corresponding to the edge jumps of Fano shapes, to be determined accurately. The resonant energy of the 3*d*_5/2_ 4*f* ^2^ state is especially strongly influenced by the Coulomb repulsion *U*_fd_ between Ce 4*f* and 5*d* electrons (Ogasawara *et al.*, 2000 ▸), which is important for elucidating the quantum critical phenomena (QCP) of valence fluctuation (Watanabe *et al.*, 2008 ▸). We can evaluate *U*_fd_ with high precision, and will make a significant contribution to the study of QCP in heavy fermions. The applications of resonant HAXPES will be greatly expanded for not only the fractured samples but also small crystalline samples ∼30 µm in size.

### 4.2. Three-dimensional spatial resolved chemical bonding state (EH2)

**Figure 12:**
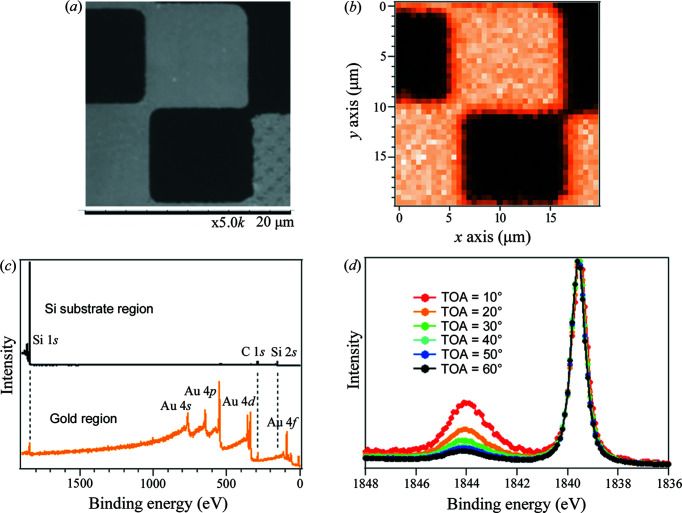
(*a*) SEM image of a 10 µm gold lattice sample on an Si substrate. (*b*) Photoelectron intensity map at the Au 4*f* core level. A checkerboard pattern image with a 10 µm interval is obtained. (*c*) Survey spectra obtained at the center of a gold square and an Si substrate surrounded by gold squares. In the Si substrate region, the gold contribution is not detected. (*d*) AR-HAXPES spectra obtained at the Si substrate region.

We performed a three-dimensional analysis of the chemical bonding state by combining the wide-angle analyzer with the 1 µm focused beam at EH2. Fig. 12 ▸(*b*) shows the Au 4*f* intensity distribution for a sample with 10 µm gold lattice on an Si substrate [Fig. 12 ▸(*a*)]. For this measurement, the sample was scanned in the two-dimensional direction along the sample surface. We confirmed that the Au 4*f* intensity correctly reflects the checkerboard pattern. Fig. 12 ▸(*c*) shows survey spectra at the center positions of a gold region and an Si substrate region surrounded by the gold regions. In the gold region, both gold-derived peaks and peaks derived from the underlying buried Si substrate are observed. On the other hand, in the Si substrate region, gold-derived peaks are not observed. Of course, there is some drift of the beam irradiation position during the measurement, but it is only a few micrometres. This drift is not due to beam position fluctuation, but instead attributed to sample position fluctuation such as thermal contraction of the instrument base. This indicates that local HAXPES analysis is available in an area of a micrometre-sized square.

Furthermore, by performing AR-HAXPES measurements in this local region, a depth dependence of chemical bonding states was obtained. The Si 1*s* AR-HAXPES spectrum at the center of the Si substrate region surrounded by the gold region is shown in Fig. 12 ▸(*d*). The spectra were acquired by integrating the angular information for ±5° of each take off angle (TOA) and normalized by the intensity of the metallic peak on the lower binding-energy side. Spectra in the small TOA region reflect the surface electronic state, while those in the larger TOA contain more information on the deep region. The AR-HAXPES spectra show that the Si substrate is naturally oxidized at the surface and the oxidation is suppressed toward the depth. The thickness of the naturally oxidized film is estimated to be 1.1 nm based on the analysis of the two-layer model using equation (1) in the work by Ikenaga *et al.* (2013 ▸). The chemical bonding state analysis in the depth direction with nanometre resolution in a local region on the micrometre scale is a unique feature of the EH2 system.

## 5. Summary

The beamline BL09XU at SPring-8 has been upgraded as a beamline dedicated for HAXPES by integrating two HAXPES activities in BL09XU and BL47XU. To perform advanced HAXPES analyses, state-of-the-art optical instruments such as DCCM, DXPR and the Wolter mirrors were installed. The instrument control system BL-774 allows us to operate all instruments seamlessly. This upgrade not only drastically improves the performance of the resonant HAXPES application and the microscale analysis of chemical bonding states but also greatly expands the possibilities of all HAXPES applications.

## Figures and Tables

**Table 1:** Specifications of the HRMs

		Bragg angle (°)	Energy (keV)	Δ*E*_M_/*h*ν (× 10^−5^)
DCCM	Si(220)	18–45	10.4–4.6	3.8
	Si(311)	18–45	12.2–5.4	1.6
CCM	Si(333)	85 (fixed)	5.95	0.75
	Si(444)	85 (fixed)	7.94	0.45
	Si(555)	85 (fixed)	9.92	0.16

**Table 2:** Operating energy ranges of DXPRs determined from conditions with the degree of polarization above 0.9 and an X-ray transmission above 0.2

Thickness (mm)	Energy (keV)
0.1	5.1–7.5
0.2	6.7–9.5
0.4	7.9–11.5

**Table 3:** Optical parameters of Wolter-type (EH1) and KB-type (EH2) focusing mirrors

	Wolter (EH1)	KB (EH2)
Focusing size (V × H) (µm)	0.5 × 26 (FE slit fully open)	0.5 × 16 (FE slit fully open)
		0.5 × 0.72 (FE width = 33 µm)
Glancing angle (mrad)	5.0, 5.0	4.5 (V, H)
Spatial acceptance (mm)	2.3 × 1.0	1.0 × 1.3
Working distance (m)	1470	840
Magnification factor	1/29	V: 1/29; H: 1/46
Cut-off energy (keV)	∼13.5	∼15.0
Surface coating	Rh	Rh

**Table 4:** Obtained focused beam size and flux in Si(311) DCCM at *h*ν = 7.94 keV by Wolter-type (EH1) and KB-type (EH2) focusing mirrors

	Wolter (EH1)	KB (EH2)	
FE slit	Fully open	Fully open	Width = 33 µm
Focused beam size (V × H) (µm)	1.3 × 21	1.5 × 11	1.3 × 0.86
Beam flux in an Si(311) DCCM at *h*ν = 7.94 keV (photon s^−1^)	5.6 × 10^12^	6.3 × 10^12^	1.3 × 10^11^
